# Proton pump inhibitor use is associated with a variety of infections in patients with liver cirrhosis

**DOI:** 10.1097/MD.0000000000023436

**Published:** 2020-12-11

**Authors:** Christian Labenz, Karel Kostev, Peter R. Galle, Marcus-Alexander Wörns, Joachim Labenz, Christian Tanislav, Charles Christian Adarkwah

**Affiliations:** a Department of Internal Medicine I, University Medical Centre of the Johannes Gutenberg-University, Mainz; b Epidemiology, IQVIA, Frankfurt am Main; c Department of Internal Medicine; d Department of Geriatrics and Neurology, Diakonie Klinikum Siegen; e Department of General Practice and Family Medicine, Philipps-University, Marburg; f CAPHRI School for Public Health and Primary Care, Department of Health Services Research, Maastricht University, Maastricht, The Netherlands.

**Keywords:** acid suppression, complications of cirrhosis, end-stage liver disease, infections, liver cirrhosis, proton pump inhibitor

## Abstract

There is evidence that intake of proton pump inhibitors (PPI) increases the risk for spontaneous bacterial peritonitis (SBP) in patients with liver cirrhosis. However, data regarding the impact of PPI intake on occurrence of infections other than SBP are still lacking.

We hypothesized that PPI use is associated with a higher rate of infections other than SBP in patients with liver cirrhosis.

The current case-control study sample included patients with liver cirrhosis from the Disease Analyzer database (IQVIA), which compiles data such as risk factors, drug prescriptions and diagnoses obtained from general practitioners and specialists in Germany. In total, 2,823 patients with infections were matched with 2,823 patients without infections by propensity scores. For quantification of PPI use the prescribed quantity of PPI during the past 12 months before index date was analyzed.

Frequency of PPI users was significantly higher in patients with infections than in patients without infections (47.9% vs 37.9%). In regression analysis, PPI use was significantly associated with the occurrence of infections overall (OR 1.55, 95% CI 1.39–1.72, *P* < .001), and associated with the occurrence of lower respiratory tract infections, urinary tract infections and infectious gastroenteritis. There was no association between PPI use and skin infections. Pantoprazole and omeprazole were the most frequently prescribed PPIs and were both independently associated with the occurrence of infections.

PPI use may be associated with infections other than SBP in patients with liver cirrhosis. Prescription of PPI should be limited to patients with a clear indication.

## 1 Introduction

Globally, liver cirrhosis is a common cause for morbidity and mortality contributing to over 1 million deaths in 2010.^[1]^ Infections in cirrhosis are not only a main cause for higher mortality but also trigger the occurrence of cirrhosis-related complications like hepatic encephalopathy or renal failure.^[2,3]^ The susceptibility of cirrhotic patients to infections results from a compromised immune system and increased bacterial translocation caused by liver insufficiency.^[4,5]^ Therefore, it is important to identify potential risk factors for the occurrence of infections in this patient population.

Proton pump inhibitors (PPIs) are yet a matter of debate. They are among the most frequently prescribed drugs in the Western World. Although cost-effectiveness has been shown when used appropriately, studies show they are very often prescribed without a clear indication, especially in the primary care setting.^[6]^

In recent years, several studies indicated that chronic intake of PPIs is associated with a higher risk for spontaneous bacterial peritonitis (SBP).^[7,8]^ This is most likely due to the fact that a disruption of the gastric acid barrier leads to a change in gut microbiota and Small Intestinal Bacterial Overgrowth (SIBO).^[9,10]^ Hence, there is evidence indicating an additional effect of PPI treatment on the risk of infections in general especially in decompensated cirrhosis.^[11,12]^ However, data regarding an additional risk of infections in different sites like the lower respiratory tract or urinary tract due to PPI use are scarce. Therefore, it was the aim of this population-based case-control study to investigate the effects of PPI use on bacterial infections other than SBP in outpatients with liver cirrhosis.

## 2 Materials and methods

### 2.1 Database

This study is based on data from the Disease Analyzer database (IQVIA), which compiles drug prescriptions, diagnoses, and basic medical and demographic data obtained directly and in anonymous format from computer systems used in the practices of general practitioners and specialists.^[13]^ Diagnoses (International Classification of Diseases, 10th revision [ICD-10]), prescriptions (Anatomical Therapeutic Chemical [ATC] Classification system), and the quality of reported data are monitored by IQVIA based on a number of criteria (e.g., completeness of documentation, linkage between diagnoses and prescriptions).

In Germany, the sampling methods used for the selection of physicians’ practices are appropriate for obtaining a representative database of general and specialized practices.^[13]^

### 2.2 Study population and variables

**Figure 1:**
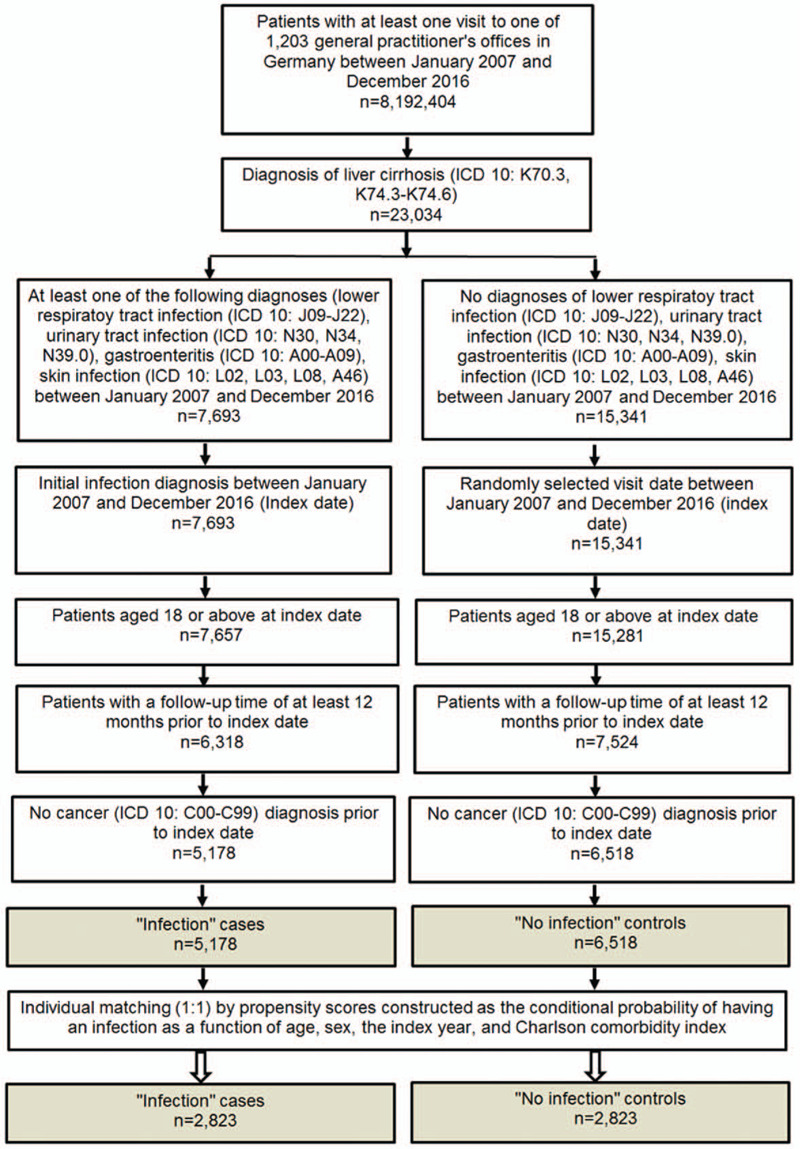
Selection of study patients.

The current study sample included patients with liver cirrhosis (International Classification of Diseases, 10th edition [ICD-10]: K74.1-K74.1) who had an infection of the lower respiratory tract (ICD-10: J09-J22), urinary tract (ICD-10: N30,N34,N39.0), infectious gastroenteritis (ICD-10: A00-A09) or a skin infection (ICD-10: L02,L03,L08,A46) between 1 January 2006 and 31 December 2015 and were followed up in one of 1.203 general practices in Germany. The date of the first injection diagnosis was considered index date. The further inclusion criteria were as follows: age ≥18 years at the index date, observation time of at least 12 months prior to index date and a follow-up time of at least 12 months after the index date. After applying inclusion criteria, patients with liver cirrhosis without infections were matched (1:1) to patients with infections based on propensity scores using a greedy algorithm and derived from the logistic regression using age, gender, index year, and Charlson comorbidity index (CCI). The Charlson index is a weighted index that accounts for the number and severity of comorbidities in administrative database studies and includes a wide range of comorbidities (macrovascular diseases, pulmonary diseases, gastrointestinal, liver and renal diseases, diabetes, tumors, and AIDS).^[14]^ The index date for the controls was a randomly selected visit between 1 January 2006 and 31 December 2015 (Fig. 1).

### 2.3 Medication exposure

PPI use included the following drugs: omeprazole (A02BC01), pantoprazole (ATC A02BC02), lansoprazole (A02BC03), rabeprazole (A02BC4O) and esomeprazole (A02BC05). Due to the small patient counts for lansoprazole, esomeprazole and rabeprazole in Germany, these 3 drugs were handled as one group (other PPIs). For quantification of PPI use the prescribed quantity of PPI (in mg) was analyzed. In this study we analyzed the PPI use in the past 12 months before the index date in the case and control cohorts. We separated PPI use into four subgroups: patients with ≤ 3000 mg per year, 3001–5000 mg, 5001–10000 mg and >10,000 mg per year.

### 2.4 Study outcome and covariables

The main outcome of the study was the risk of infection other than SBP as a function of PPI use. Covariables included age, sex, CCI, liver cirrhosis diagnosis detail including alcoholic cirrhosis of liver (ICD-10: K70.3), primary biliary cirrhosis (ICD-10: K74.3), secondary biliary cirrhosis (ICD-10: K74.4), biliary cirrhosis, unspecified (ICD-10: K74.5), unspecified cirrhosis of liver (ICD-10: K74.6), and hepatic decompensation at index date including ascites (ICD-10: R18), history of variceal bleeding (ICD-10: I86.4) history of hepatorenal syndrome (ICD-10: K76.7), and history of hepatic encephalopathy (ICD-10: K72.9). As complications of liver cirrhosis are not routinely documented and coded in GP practices, medication usually prescribed to treat these complications, were included in the analyses: that is, propranolol and carvedilol for treatment of esophageal varices, rifaximin and lactulose for treatment of hepatic encephalopathy and norfloxacin for treatment status post spontaneous bacterial peritonitis.

### 2.5 Ethic statement

This study was conducted according to the ethical guidelines of the 1975 Declaration of Helsinki (6th revision, 2008). German law allows the use of anonymous electronic medical records for research purposes under certain conditions. According to this legislation, it is not necessary to obtain informed consent from patients or approval from a medical ethics committee for this type of observational study that contains no directly identifiable data. Because patients were only queried as aggregates and no protected health information was available for queries, no institutional review board (IRB) approval was required for the use of this database or the completion of this study.

### 2.6 Statistical analysis

Descriptive analyses were obtained for all demographic and clinical variables, and differences between cases and controls were evaluated using Wilcoxon signed-rank test for continuous variables and chi-squared test for categorical variables. Two logistic regression models were conducted to study the association between the use of PPI in total, as much as pantoprazole, esomeprazole and other PPIs separately, and the infection risk. The first model based on non-matched patients and was adjusted for sex, age, CCI, liver cirrhosis diagnosis detail, and hepatic decompensation at index date. The second model based on matched-pairs and was adjusted for liver cirrhosis diagnosis detail, and hepatic decompensation at index date. Bonferroni correction was carried out to counteract the problem of multiple comparisons. With 12 variables in the second regression model, *P* values < .004 (calculated as 0.05/12) were considered statistically significant. For other analyses a *P* value of < .05 was considered statistically significant. The statistical analyses were performed with SAS 9.4.

## 3 Results

### 3.1 Basic characteristics of the study sample

The selection of the study patients is displayed in Figure 1. The present study included 5,178 patients with liver cirrhosis with infections and 6,518 patients with liver cirrhosis without infections. After individual matching by propensity scores as a function of age, sex, index year and Charlson Comorbidity index 2 groups (each n = 2.823) of cirrhotic patients with and without infections remained. The baseline characteristics of study patients are displayed in Table 1.

**Table 1:** Baseline characteristics in cirrhotic patients with and without infections.

	Prior to 1:1 matching			After the 1:1 match		
	Patients with infection (n = 5,178)	Patients without infection (n = 6,518)	*P* value	Patients with infection (n = 2,823)	Patients without infection (n = 2,823)	*P* value
Sex						
male	55.0	62.6	<.001	59.8	59.8	1.000
female	45.0	37.4		40.2	40.2	
Age at index date (mean, SD)	62.6 (12.2)	62.4 (12.4)	.135	62.5 (11.5)	62.5 (11.5)	1.000
Charlson Comorbidity Index 12 months prior to index date (SD)	4.3 (2.2)	3.3 (2.1)	<.001	3.7 (1.8)	3.7 (1.8)	1.000
Liver cirrhosis diagnosis detail (%)						
Alcoholic cirrhosis of liver (K70.3)	18.4	21.9	<.001	18.9	22.4	.001
Primary biliary cirrhosis (K74.3)	7.7	4.6	<.001	7.4	4.9	.001
Secondary biliary cirrhosis (K74.4)	0.6	0.4	.162	0.3	0.3	1.000
Biliary cirrhosis, unspecified (K74.5)	1.9	0.8	<.001	1.7	1.0	.016
Unspecified cirrhosis of liver (K74.6)	71.5	72.2	.389	71.7	71.5	.860
Complications of disease (%)						
Treatment of esophageal varices with propranolol or carvedilol	17.7	15.5	.002	16.4	16.2	.857
Treatment of hepatic encephalopathy with rifaximin and/or lactulose	11.4	10.1	.017	10.7	10.7	.931
Treatment with norfloxacin for s/p spontaneous bacterial peritonitis	1.1	0.3	<.001	1.1	0.2	<.001
Hepatic decompensation at index date, n (%)						
Ascites (R18)	9.4	8.7	.174	9.0	8.9	.816
History of variceal bleeding (I86.4)	0.3	0.2	.329	0.1	0.2	.317
History of Hepatorenal syndrome (K76.7)	0.6	0.3	.018	0.6	0.1	.007
History of hepatic encephalopathy (K72.9)	3.8	3.0	.023	2.7	3.2	.307

SD = standard deviation.

### 3.2 Association between PPI use and risk for infections other than SBP

In the total cohort 50.0% of the patients with infections and 33.7% of the patients without infections were PPI users. Additionally, 14.6% of the patients with infections and 8.9% of the patients without infections had an intake of more than 10,000 mg PPI per year prior to the index date (Table 2). Regression analysis showed, that PPI use was significantly associated with the occurrence of infections in the unmatched cohort (OR 1.62, 95% CI 1.49–1.75, *P* < .001) as well as in the propensity matched cohort (OR 1.55, 95% CI 1.39–1.72, *P* < .001). This association remained significant independent of the cumulative PPI dose during the year prior to the infections (each *P* < .001, Table 2).

**Table 2:** Association between proton pump inhibitor use and risk for infections other than spontaneous bacterial peritonitis stratified for infect localization in patients followed by general practitioners in Germany.

	Regression analysis based on non-matched patients, adjusting for sex, age, CCI, liver cirrhosis diagnosis details, therapy of liver cirrhosis complications, and hepatic decompensation at index date				Regression analysis based on matched pairs, adjusting for liver cirrhosis diagnosis details, therapy of liver cirrhosis complications, and hepatic decompensation at index date			
	Proportion in patients with infection	Proportion in patients without infection	OR (95% CI)	*P* value	Proportion in patients with infection	Proportion in patients without infection	OR (95% CI)	*P* value
All infections	n = 5,178	n = 6,518			n = 2,823	n = 2,823		
Any PPI	50.0	33.7	1.68 (1.55–1.82)	<.001	47.9	37.9	1.55 (1.39–1.72)	<.001
≤ 3000 MG	13.8	11.0	1.57 (1.39–1.77)	<.001	13.8	12.1	1.38 (1.18–1.63)	<.001
3001–5000 MG	7.2	5.1	1.63 (1.38–1.92)	<.001	7.2	5.5	1.60 (1.28–2.00)	<.001
5001–10,000 MG	14.4	8.7	1.82 (1.60–2.07)	<.001	13.5	10.1	1.63 (1.38–1.93)	<.001
>10,000 MG	14.6	8.9	1.72 (1.51–1.95)	<.001	13.5	10.2	1.63 (1.38–1.93)	<.001
Lower respiratory tract	n = 1,933	n = 6,518			n = 1,086	n = 1,086		
Any PPI	48.5	33.7	1.64 (1.46–1.83)	<.001	46.0	37.9	1.48 (1.24–1.76)	<.001
≤ 3000 MG	13.1	11.0	1.51 (1.29–1.78)	<.001	13.6	12.0	1.28 (0.99–1.66)	.060
3001–5000 MG	6.7	5.1	1.52 (1.22–1.91)	<.001	6.5	6.6	1.19 (0.83–1.68)	.343
5001–10,000 MG	12.9	8.7	1.64 (1.38–1.95)	<.001	11.3	9.0	1.53 (1.14–2.04)	.004
>10,000 MG	15.9	8.9	1.88 (1.59–2.23)	<.001	14.5	9.5	1.90 (1.44–2.50)	<.001
Urinary tract	n = 1,081	n = 6,518			n = 550	n = 550		
Any PPI	53.3	33.7	1.77 (1.54–2.04)	<.001	51.8	35.5	1.95 (1.52–2.50)	<.001
≤ 3000 MG	15.0	11.0	1.77 (1.44–2.18)	<.001	15.5	9.1	2.21 (1.50–3.26)	<.001
3001–5000 MG	8.9	5.1	1.99 (1.53–2.59)	<.001	8.4	4.2	2.65 (1.56–4.50)	<.001
5001–10,000 MG	15.1	8.7	1.77 (1.42–2.19)	<.001	14.2	12.9	1.47 (1.02–2.12)	.039
>10,000 MG	14.3	8.9	1.64 (1.32–2.04)	<.001	13.8	9.3	2.04 (1.37–3.03)	<.001
Infectious gastroenteritis	n = 1,215	n = 6,518			n = 523	n = 523		
Any PPI	50.6	33.7	1.81 (1.58–2.07)	<.001	49.6	37.7	1.68 (1.34–2.10)	<.001
≤ 3000 MG	15.4	11.0	1.77 (1.46–2.13)	<.001	15.1	12.7	1.51 (1.09–2.10)	.013
3001–5000 MG	7.0	5.1	1.65 (1.27–2.15)	<.001	7.5	5.0	1.92 (1.20–3.09)	.007
5001–10,000 MG	14.1	8.7	1.94 (1.58–2.38)	<.001	13.7	9.3	1.91 (1.34–2.73)	<.001
>10,000 MG	14.2	8.9	1.82 (1.49–2.25)	<.001	13.3	10.7	1.57 (1.11–2.23)	.012
Skin infection	n = 949	n = 6,518			n = 523	n = 523		
Any PPI	48.7	33.7	1.47 (1.27–1.70)	<.001	45.9	40.7	1.22 (0.95–1.56)	.123
≤ 3000 MG	12.0	11.0	1.25 (1.00–1.56)	.054	10.9	13.2	0.87 (0.59–1.28)	.482
3001–5000 MG	6.9	5.1	1.37 (1.03–1.84)	.033	6.9	5.2	1.48 (0.87–2.51)	.145
5001–10000 MG	16.9	8.7	1.95 (1.58–2.40)	<.001	16.8	10.5	1.74 (1.19–2.54)	.004
>10,000 MG	13.0	8.9	1.45 (1.07–1.69)	.019	11.3	11.9	1.03 (0.69–1.53)	.881

CCI = Charlson Comorbidity Index, CI = confidence interval, mg: milligram, OR = odds Ratio, PPI = proton pump inhibitor.

To investigate the association of PPI use and various types of infections different regression models were run. For instance, PPI use remained significantly associated with the occurrence of infections of the lower respiratory tract, the urinary tract as well as the occurrence of gastroenteritis in the unmatched as well as in the matched cohorts (each *P* < .001). In the unmatched cohort PPI use and especially doses higher than 3000 mg per year were significantly associated with skin infections. However, this association could not be observed in the propensity matched cohort (*P* = .123, Table 2). The association of different cumulative PPI doses and different types of infections is also presented in Table 2.

### 3.3 Association between type of PPI and risk for infections other than SBP

Pantoprazole followed by omeprazole was the most frequently used PPI in this German cohort. To investigate the association between the used type of PPI and the occurrence of infections separate models were run for pantoprazole, omeprazole and “other PPIs”, as further described above (Table 3). Here, pantoprazole (OR 1.54, 95% CI 1.36–1.74, *P* < .001) and omeprazole (OR 1.63, 95% CI 1.38–1.94, *P* < .001) were significantly associated with infections in the unmatched as well as in the propensity matched cohort. Other PPI use was only associated to the occurrence of infections in the unmatched cohort, while there was only a trend in the propensity matched cohort (OR 1.65, 95% CI 1.12–2.42, *P* = .011). Impact of different doses of PPIs on the presence of infections are displayed in Table 3.

**Table 3:** Association between proton pump inhibitor use and risk for infections other than spontaneous bacterial peritonitis stratified for type of PPI in patients followed by general practitioners in Germany.

	Regression analysis based on non-matched patients, adjusting for sex, age, CCI, liver cirrhosis diagnosis detail, and hepatic decompensation at index date				Regression analysis based on matched pairs, adjusting for liver cirrhosis diagnosis detail, and hepatic decompensation at index date			
	Proportion in patients with infection	Proportion in patients without infection	OR (95% CI)	*P* value	Proportion in patients with infection	Proportion in patients without infection	OR (95% CI)	*P* value
Pantoprazole	40.5	25.5	1.59 (1.45–1.74)	<.001	38.1	29.4	1.54 (1.36–1.74)	<.001
≤ 3000 MG	10.3	8.2	1.41 (1.22–1.62)	<.001	10.0	9.1	1.28 (1.05–1.56)	.014
3001–5000 MG	6.0	3.7	1.63 (1.34–1.99)	<.001	5.9	4.6	1.54 (1.19–2.01)	.001
5001–10000 MG	11.1	6.2	1.76 (1.51–2.05)	<.001	10.2	7.2	1.71 (1.39–2.10)	<.001
>10000 MG	13.1	7.4	1.62 (1.41–1.87)	<.001	12.0	8.6	1.68 (1.38–2.05)	<.001
Omeprazole	21.1	12.3	1.59 (1.40–1.80)	<.001	20.8	14.2	1.63 (1.38–1.94)	<.001
≤ 3000 MG	6.9	4.1	1.76 (1.43–2.15)	<.001	7.1	4.8	1.63 (1.24–2.14)	<.001
3001–5000 MG	2.8	1.9	1.33 (0.98–1.80)	.070	2.9	1.8	1.73 (1.13–2.66)	.012
5001–10000 MG	6.8	3.7	1.66 (1.34–2.05)	<.001	6.4	4.6	1.58 (1.19–2.09)	.002
>10000 MG	4.6	2.7	1.44 (1.13–1.85)	.004	4.5	3.1	1.67 (1.19–2.34)	.003
Other PPI	5.2	2.3	1.90 (1.45–2.50)	<.001	4.2	2.7	1.65 (1.12–2.42)	.011
≤ 3000 MG	1.5	0.8	1.85 (1.17–2.94)	.009	1.4	1.0	1.48 (0.79–2.77)	.226
3001–5000 MG	0.9	0.5	1.68 (0.91–3.12)	.097	0.7	0.3	2.84 (0.98–8.28)	.055
5001–10000 MG	1.4	0.6	1.94 (1.14–3.29)	.015	1.2	0.8	1.67 (0.83–3.36)	.150
>10000 MG	1.4	0.5	2.15 (1.23–3.76)	.007	0.9	0.6	1.38 (0.61–3.15)	.440

CCI = Charlson Comorbidity Index, CI = confidence interval, mg: milligram, OR = odds Ratio, PPI = proton pump inhibitor.

## 4 Discussion

Infections are a leading factor for increased mortality in patients with cirrhosis and may cause decompensation or acute-on-chronic liver failure. In this current study, we could demonstrate that PPI use may be associated with the occurrence of gastroenteritis, lower respiratory and urinary tract infections in patients with liver cirrhosis, while there seems to be no association with the occurrence of skin infections. The association described above was independent of the PPI administered.

PPI are among the most prescribed medications worldwide. In our population of cirrhotic patients treated in primary care, 50.0% of patients of the infection cohort and 33.7% of the patients without infections were treated with PPI during the previous year. This is in line with other studies investigating cohorts from Europe. A recently published study by Dam et al, which analysed data from the satavaptan trials, showed that 54% of their patients with decompensated cirrhosis were on PPI treatment.^[11]^ In other studies prevalence of PPI intake was even as high as 78.3%.^[15]^ Hence, this is a remarkable finding since evidence suggests that more than half of the patients have no indication for a permanent PPI therapy.^[16,17]^

In our study, we found that PPI use is associated with infections other than SBP except for skin infections. There are only a few studies, which investigated the impact of PPI use on infections other than SBP and are therefore comparable to ours. Data from a recently published study by Dam et al are more or less in line with our findings and validate the generalizability of our relative risk estimates.^[11]^ Here, the authors analysed data from the satavaptan trials and found that PPI use is associated with SBP (HR 1.74), urinary tract infections (HR 1.98), and gastroenteritis (HR 2.30) while there was no association with skin infections (HR 1.32, 95% CI 0.65–2.66). An association between PPIs and lower respiratory tract infection (OR 1.58, 95% CI 0.94–2.65) showed a clear trend but did not reach significance. Although they included a large number of patients into their study, this finding is most likely explained by a type II error due to a low incidence of lower respiratory tract infections (n = 63) in their cohort. Due to our study design we were able to analyse data of a huge cohort of 1.086 patients with lower respiratory tract infections leading to robust results in this subgroup. Another study by Merli et al found an association between PPI use and bacterial infections in a cross-sectional study including 400 hospitalized patients from Italy.^[17]^ However, in contrast to our study, they found no association between PPI use and the site of infection. This is most likely explained by the fact that their study was not powered to address this issue in detail.

To the best of our knowledge, this is the first study to investigate the association of different types of PPI and occurrence of infections other than SBP in cirrhotic patients. Pantoprazole as well as omeprazole seemed to be associated with infections while there was only a trend in the propensity matched cohort for other PPIs. The fact that other PPIs than pantoprazole and omeprazole did not reach statistical significance in our propensity matched model is most likely explained by the relatively small patient counts for lansoprazole, esomeprazole and rabeprazole in Germany, which were summarized under other PPIs.

There are several explanations for our findings that PPI use increases the risk for especially infectious gastroenteritis, lower respiratory and urinary tract infections. First, it is a known fact that PPI uses decreases the gastric pH barrier and consequently promotes proliferation of different bacteria.^[9,18,19]^ Additionally, there are data on the proliferation of oral Streptococcaceae in the stomach after PPI treatment. This may also result in bacterial translocation through micro-aspiration of gastric fluid and could consequently lead to the development of lower respiratory infections.^[18,20,21]^ Second, infections on different sites like the urinary tract may be caused by bacterial translocation.^[22]^ This translocation is associated with the presence of small intestinal bacterial overgrowth which can be triggered by PPIs.^[23,24]^ Finally, studies indicated that PPIs may have an adverse effect on anti-inflammatory components by affecting innate immunity and neutrophil functioning.^[25]^

Strength of this study is that it examines information from a highly representative computerized population-based database and includes a large sample size. Nevertheless, our study has some limitations inherent to database analysis research. The conducted analysis relies on ICD-10 codes for establishing diagnoses. This may cause misclassification bias due to miscoding or under coding of diagnoses. In our study more than 70% of the patients were not coded with the respected aetiology of their liver cirrhosis. This is most likely explained by the fact that coding of aetiologies of cirrhosis does not result in a higher reimbursement in Germany. However, we believe that this may not bias our findings and the reliability of the German Disease Analyzer database has been validated in several medical studies.^[13]^ Furthermore, it has to be mentioned that the German Disease Analyzer database does not capture detailed laboratory values. Therefore, our current study lacks information regarding disease severity as expressed by MELD or Child-Pugh score. Nevertheless, we tried to adjust our regression models for complications of cirrhosis and typical cirrhosis medications like non-selective beta-blockers or lactulose and believe that a potential bias may be negligible. Our estimations of PPI use in mg per year prior to index date was derived from filled prescriptions. These data may not reflect the actual dose taken by the patients. On the one hand it seems possible that patients did not take every pill as prescribed and on the other hand some patients may have bought PPIs without prescription. However, this bias may be present in patients with as well as patients without infections and therefore negligible. Last, the Disease Analyzer database^[13]^ is an outpatient database. As a result, it is inherently incapable of capturing patients who suffered from infections and died during the same hospital stay. This may result in a cohort with focus on earlier stages of liver cirrhosis and less severe infections.

In conclusion, our study demonstrates that PPI use may be associated with infectious gastroenteritis, lower respiratory and urinary tract infections in patients with cirrhosis, while there seems to be no associations with skin infections. This association was independent of the used PPI. Therefore, in the management of patients with liver cirrhosis the indication regarding prescription of PPI should be questioned critically.

## Author contributions

**Contributed reagents/materials/analysis tools:** Karel Kostev

**Designed the experiments and analysed the data:** Christian Labenz, Karel Kostev, Peter R. Galle, Marcus-Alexander Wörns, Joachim Labenz, Christian Tanislav, Charles Christian Adarkwah

**Guarantor of the article:** Charles Christian Adarkwah

**Performed research:** Christian Labenz, Karel Kostev, Peter R. Galle, Marcus-Alexander Wörns, Joachim Labenz, Christian Tanislav, Charles Christian Adarkwah

**Statistical analysis:** Karel Kostev

**Wrote the paper:** Christian Labenz, Karel Kostev, Charles Christian Adarkwah
